# Wolff-Parkinson-White Syndrome and Rheumatic Mitral Stenosis: an Uncommon Coincidence that can Cause Severe Hemodynamic Disturbance

**Published:** 2008-11-01

**Authors:** Ahmet Taha Alper, Hakan Hasdemir, Ahmet Akyol

**Affiliations:** Siyami Ersek Thoracic and Cardiovascular Surgery, Center, Cardiology Department Istanbul, Turkey

**Keywords:** Wolff-Parkinson-White Syndrome, Rheumatic Mitral Stenosis

## Abstract

The combination of rheumatic mitral stenosis and Wolff-Parkinson-White syndrome is a rare situation. In this case, we are reporting an 72-year-old man presenting with multi-organ failure due to the this combination and successfully treated with radiofrequency ablation during preexcitated atrial fibrillation.

## Introduction

The combination of Wolff-Parkinson-White (WPW) syndrome and rheumatic mitral stenosis was rarely reported in the literature1-3. In our case report, we present a patient with multi- organ failure due to incessant preexcited atrial fibrillation (AF) triggered by this combination and treated with radiofrequency ablation during tachycardia.

## Case report

**Figure 1:**
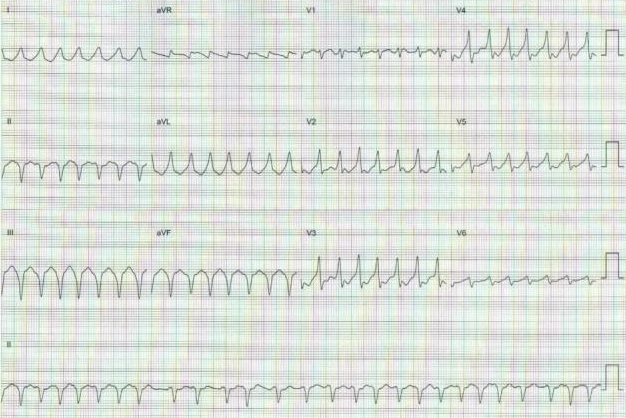
Twelve-lead ECG showing preexcitated atrial fibrillation

**Figure 2:**
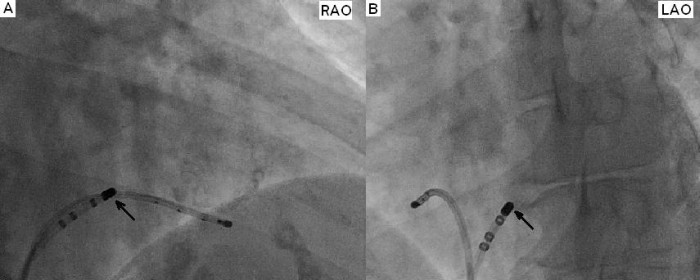
***a, b*** Ablation site (arrow) in the right and left anterior oblique views (RAO, LAO).

**Figure 3:**
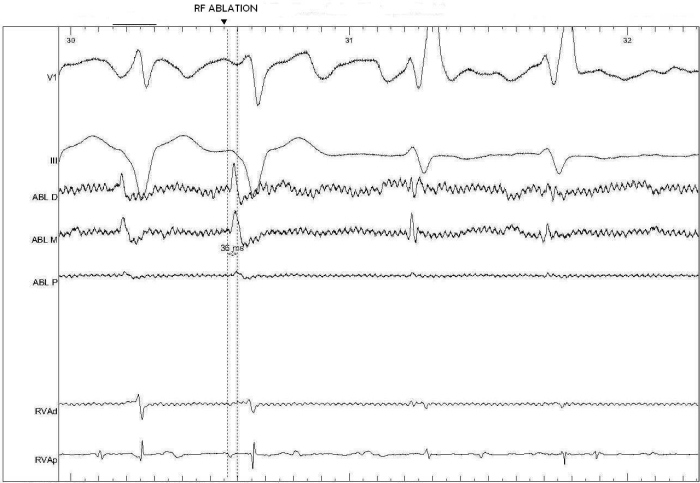
Recordings at the site of successful ablation. Earliest ventricular activation precedes the onset of QRS by 36 ms in the distal bipolar recordings. ABL, ablation catheter;  RV, right ventricle

A 72-year-old man presented to the emergency department with the complaint of palpitation, progressive dyspnea and mental status deterioration present for four days. Physical examination revealed a blood pressure of 70/40 mmHg and an irregular heart rate of 190-240 beats per minute. The 12 lead- electrocardiogram showed preexcited atrial fibrillation (Figure 1). Urgent cardioversion was required. So transesophageal echocardiography was performed to rule out the presence of left atrial thrombus. Echocardiography revealed a mitral valve area of 1.19 cm^2^ with a gradient of 20/10 mmHg, moderate tricuspid regurgitation and no left atrial thrombus. A biochemical profile obtained on admission revealed high levels of serum glutamic-oxaloacetic acid transferase (SGOT), serum glutamic pyruvic transaminase (SGPT), international normalized ratio (INR) and creatinine (CR) (SGOT 1800 IU/L, SGPT 3600 IU/L, INR 6.3 CR: 2.8 mg/dl) indicating hepatic and renal failure. Because of hemodynamic instability, immediate cardioversion was performed. But multiple attempts of electrical cardioversion using biphasic energy failed to restore normal sinus rhythm. Also it was not possible to control the ventricular rate medically. Because of hepatic failure, amiodarone couldn't be given to control the ventricular rate. After obtaining informed consent the patient was transferred to the electrophysiology laboratory. Because stable cannulation of coronary sinus was not possible due to anatomical reasons, two electrode catheters, one 6-French Josephson catheter, and one 7-French ablation catheter (Medtronic, Minneapolis, MN), were advanced through both femoral veins and placed at right ventricular apex, and tricuspid annulus positions. During AF, full preexcitation was present in 12-lead ECG. According to 12-lead ECG, accessory pathway was right paraseptal in location. But to exclude the possiblity of a left-sided paraseptal accessory pathway, coronary sinus was mapped by the ablation catheter. Mapping of coronary sinus revealed that earliest ventricular activation in coronary sinus was located in the ostium. Then, endocardial mapping of the tricuspid annulus during preexcited AF showed the area of earliest ventricular activation at the level of the right midseptal region (Figure 2: ***a, b***). Current was delivered at this site using automatic temperature control with a maximum power setting of 30 watt and a target temperature of 50ºC. Because it was impossible to follow 1:1 AV conduction during ablation, we only observed bradycardia and regularity of the rhythm to follow AV conduction. Radiofrequency current was then applied abolishing the preexcitation in the first second of the application without bradycardia and regularity of AF (Figure 3).  Then after 20 seconds, setting of the generator was changed to a maximum of 50 watt and a target temperature of 60ºC. Ablation was performed for total of 60 seconds. After ablation, AF was still present, but the rate was less than 100 bpm and QRS complexes were narrowed. No preexcitation was seen and there was no AV block. After ablation procedure, electrical cardioversion was reattempted and sinus rhythm was restored. But after 1 hour, atrial fibrillation resumed again. Because mitral stenosis was present and the recurrence  risk of AF was high after even a successful cardioversion and rate control was perfect after ablation of accessory pathway, cardioversion was not considered. After ablation, rate control was obtained by AV nodal blocking agents easily. SGOT, SGPT, INR and CR levels reduced to normal range after sixth day of ablation.

## Discussion

This is the first case in literature in which RF ablation was performed during preexcited atrial fibrillation triggered by WPW syndrome and mitral stenosis. The combination of WPW syndrome and mitral stenosis was rarely reported in literature [1-3]. Similar to our case, the main point in these cases was this combination may lead to preexcited atrial fibrillation refractory to medical therapy [2,3]. In some of these cases, simultaneous  RF ablation and surgery was performed, while in some ablation was performed prior to surgery [3,4]. The main cause which made us not to prefer surgery was the appearance and progression of multiorgan failure due to incessant tachycardia. The possible explanation of this situation was high ventricular conduction rates in the presence of mitral stenosis reduced ventricular filling to a point that pump failure ensued. This explanation was supported by the fact that SGOT, SGPT, INR and CR values returned to normal values five days after ablation.

In our case, ablation was performed during preexcited atrial fibrillation due to incessant tachycardia which was resistant to electrical cardioversion. In literature, limited number of cases were reported in whom ablation during preexcited atrial fibrillation in the presence of right-sided accessory pathway was performed. Recently, Kose et al. studied long term results of patients ablated during sinus rhythm and during preexcited atrial fibrillation [5]. In this study, ablation was performed during preexcited atrial fibrillation in the presence of right sided accessory pathway in eight patients. Long term success rates of these patients were found similar to patients ablated while in sinus rhythm. No recurrence of preexcitation has been observed in our patient during six months of follow up.

Main problem during ablation of paraseptal accessory pathways during preexcited AF is the difficulty of observing the AV nodal conduction. Until now, no method for observing AV nodal conduction has been described during ablation of accessory pathway in the presence of AF [5]. So, we only followed the regularity of the rhythm and bradycardia during ablation to observe AV nodal conduction. Also at the begining, we decreased the degree and power values during the ablation. After seeing the disappearence of accesory pathway and normal AV nodal conduction, we increased these values to the usual level. As the patient health status progressively worsened, we explained the risks of AV block to the patient and patient accepted the risk of AV block and pacemaker implantation.

## Conclusion

As a result, we suggest that ablation therapy of  accessory pathway is a feasible option in patients with rheumatic MS and WPW syndrome, presenting with preexcited atrial fibrillation refractory to cardioversion and  leading to multi-organ dysfunction.
